# Anti-NMDA receptor encephalitis and psychosis: case report and literature review

**DOI:** 10.1192/j.eurpsy.2022.2247

**Published:** 2022-09-01

**Authors:** T. Gutierrez Higueras, B. Hernández Gajate, R.M. Fiestas Velasco, F. Calera Cortés, S. Sainz De La Cuesta Alonso, S. Vicent Forés, M. Reyes Lopez

**Affiliations:** 1 Reina Sofia University Hospital, Psychiatry, Córdoba, Spain; 2 Hospital Reina Sofía Córdoba, Psychiatry, Cordoba, Spain; 3 Hospital Reina Sofía Córdoba, Psychiatry, Córdoba, Spain

**Keywords:** Treatment, Encephalitis, Psychosis, Anti-NMDA

## Abstract

**Introduction:**

Anti-NMDA receptor encephalitis is a disease occurring when antibodies produced by the body’s own immune system attack NMDA receptors in the brain. Their functions are critical for judgement, perception of reality, human interaction, the formation and retrieval of memory, and the control of autonomic functions. The objective of treatment is to reduce the levels of antibodies in the blood and spinal fluid. Treatments include corticosteroids, intravenous immunoglobulin and plasmapheresis in addition to other immunomodulators, such as cyclophosphamide or rituximab.

**Objectives:**

To present a case of a 64 year-old patient who came to the emergency service of our hospital with long-standing anxiety, irritability, recurrent amnestic failures, visual hallucinations and recent-onset episodes of aggressiveness with his family. He required admission to the psychiatry department and was finally diagnosed with autoimmune anti-NMDA encephalitis by detecting antibodies in blood and CSF.

**Methods:**

Clinical case presentation and literature review of cases, focusing on psychotic symptoms.

**Results:**

A 65-year-old patient who was being studied by neurology and psychiatry departments for cognitive impairment and psychotic symptoms was admitted to Neurology after a positive lumbar puncture result for NMDA antibodies.During admission, the patient continued with a significant behavioral alteration that gradually remitted with the use of Quetiapine, corticosteroids and rituximab.

**Conclusions:**

NMDA-encephalitis has a highly variable clinical presentation, which can lead to confusion with infectious etiology or psychiatric disorders, making the diagnosis difficult, which is only possible by detecting anti-NMDA antibodies in CSF. Recognition of the disease and coordination between services is essential for early diagnosis and treatment.

**Disclosure:**

No significant relationships.

